# Effectiveness of Peritoneal Lavage in Reducing Postoperative Pain in Elective Laparoscopic Cholecystectomy: A Prospective Cohort Study

**DOI:** 10.3390/medsci14020287

**Published:** 2026-06-03

**Authors:** Aurelio Arturo Orellana Vicuña, Percy Soto Becerra, Alexis Germán Murillo Carrasco, Daysi Zulema Díaz-Obregón

**Affiliations:** 1 Postgraduate School, Universidad Peruana Cayetano Heredia, Lima 15102, Peru; aurelio.orellana@upch.pe (A.A.O.V.); daysiz.diaz.o@gmail.com (D.Z.D.-O.); 2 Centro de Innovación e Investigación Traslacional en Salud, Universidad Privada del Norte, Lima 15072, Peru; 3 Immunology and Cancer Research Group-IMMUCA, Organization for Medical Innovation and Collaboration for Science (OMICS), Lima 15001, Peru; 4 ONG Innovation and Science for the Care and Support of Society—INNOVACARE, Lima 15023, Peru

**Keywords:** laparoscopic cholecystectomy, peritoneal lavage, postoperative pain

## Abstract

Introduction: Postoperative pain in laparoscopic cholecystectomy is not well studied. Peritoneal lavage is performed in cases of acute or complicated cholecystitis, whereas it is uncommon in cases considered clean surgeries; paradoxically, this latter group reports more pain. Objective: To evaluate the association between peritoneal lavage and postoperative pain levels in patients undergoing laparoscopic cholecystectomy. Methods: Prospective cohort study on 110 patients with gallbladder pathology scheduled for elective laparoscopic cholecystectomy. Groups: with peritoneal lavage (n1 = 55) and without peritoneal lavage (n2 = 55). Pain was evaluated at 6, 12, and 24 h using the visual analog scale (VAS). Numeric variables were described as means or medians. Categorical variables were described as absolute frequencies and percentages. The association between peritoneal lavage and postoperative pain levels was analyzed using Linear Quantile Mixed Models (LQMMs) with the ‘lqmm’ package in R. Results: Pain assessment shows that the median pain scale scores were significantly lower at 6, 12, and 24 postoperative hours (*p* < 0.001) in patients who received peritoneal lavage. The adjusted median differences ranged from −1.6 to −1.7 points on the VAS across all evaluated time points, indicating consistently lower postoperative pain scores in the lavage group. Conclusions: Peritoneal lavage with saline solution during laparoscopic cholecystectomy was associated with lower postoperative pain scores during the first 24 postoperative hours.

## 1. Introduction

Gallstone disease is highly prevalent worldwide, particularly in Western countries, with lower rates observed in Eastern and African regions. North American Indigenous populations exhibit the highest prevalence (29.5% in men and 64.1% in women), followed by Italians (9%), and Germans and Japanese (7.5%) [1]. In Peru, the prevalence ranges between 10% and 21%, making laparoscopic cholecystectomy a common surgical procedure [2].

Postoperative pain is a frequent outcome in surgeries and is influenced by multiple factors, such as the surgical wound, the type of surgery, and the techniques used. Laparoscopic surgeries tend to result in less postoperative pain compared to conventional surgeries due to smaller incisions and less invasive techniques, whereas traditional surgeries require larger incisions and more complex sutures [3,4].

In laparoscopic cholecystectomy, several factors contribute to postoperative pain, including the use of electrocautery, the underlying gallbladder pathology, and the pneumoperitoneum. During surgery, dissection is typically performed using a dissector hook with monopolar energy in coagulation mode, which causes burns in the gallbladder bed. Some centers prefer using ultrasound dissection to minimize this effect [5]. Additionally, peritoneal lavage is often used to treat burns and reduce peritoneal irritation, especially in emergency cases such as acute cholecystitis and other complications that increase peritoneal secretions [5,6].

Pneumoperitoneum itself is a significant factor, with variables such as the volume and temperature of the gas, intra-abdominal pressure, and the duration of the procedure potentially causing shoulder pain due to phrenic nerve stimulation [7,8]. Peritoneal biopsies following laparoscopy have revealed peritoneal inflammation as well as capillary and nerve ruptures. Animal studies have shown that peritoneal acidosis, resulting from carbonic acid formed by CO_2_ insufflation, can be mitigated by peritoneal lavage, which reduces this pain-inducing factor [9].

Various techniques have been explored to reduce postoperative pain in laparoscopic cholecystectomy, including local anesthetic infiltration at surgical ports, peritoneal lavage with local anesthetics, and different forms of regional anesthesia [10,11]. However, there is no clear consensus on the most effective method. The use of saline solution for perihepatic peritoneal lavage is a commonly discussed practice, though it is poorly documented in the literature [10].

Some studies suggest that peritoneal lavage with isotonic saline at the end of laparoscopic cholecystectomy may help reduce postoperative pain, enabling effective pain control with standard analgesics while minimizing the need for opioid use or combined analgesic therapies [12,13]. This effect is thought to result from a reduction in CO_2_ absorption during laparoscopic procedures [14]. Additionally, the saline solution may act directly on burns in the gallbladder bed and help remove debris, such as tissue, blood, and bile, which reduces peritoneal irritation and facilitates the removal of carbonic acid generated by CO_2_ from the pneumoperitoneum [15,16].

Importantly, recent evidence-based guidelines, such as the PROSPECT review, recommend saline peritoneal lavage followed by suction as part of multimodal strategies to reduce postoperative pain after laparoscopic cholecystectomy; however, these recommendations are derived from heterogeneous studies and are not specifically focused on their isolated effect in an elective, clean surgical setting [17]. Furthermore, most available studies and guidelines emphasize overall analgesic protocols or shoulder pain related to pneumoperitoneum, rather than abdominal postoperative pain as a primary outcome. In clinical practice, peritoneal lavage is predominantly applied in acute or complicated cases, while its role in elective laparoscopic cholecystectomy remains underexplored and inconsistently implemented. Therefore, a significant gap persists regarding the magnitude and consistency of its analgesic effect in this specific context.

Perihepatic peritoneal lavage is especially important in cases of acute cholecystitis, pyocolecystitis, necrosis, and gallbladder perforation [6]. However, it is often considered unnecessary in clean procedures, such as gallbladder polyps or chronic calculous cholecystitis. Many surgeons do not routinely perform this practice, citing reasons such as lack of awareness, time constraints, or underestimation of its potential benefits in reducing postoperative pain [2].

The primary objective of this study was to evaluate whether peritoneal lavage with isotonic saline at the end of laparoscopic cholecystectomy can effectively reduce postoperative pain and minimize the need for opioids or additional analgesics in patients.

## 2. Materials and Methods

### 2.1. Study Type

This study is a prospective, analytical, observational cohort study conducted with adult patients who underwent elective laparoscopic cholecystectomy at the Department of General and Digestive Surgery of the Edgardo Rebagliati Martins National Hospital, EsSalud, between July and December 2018. The corresponding STROBE checklist is provided in Supplementary Table S1.

### 2.2. Sample Size Calculation

Initially, a pilot study was conducted, authorized by the head of Surgery Service 3AII, in which 20 clinical records of previously operated patients were anonymously reviewed. Postoperative pain intensity was analyzed 6 h after surgery using the visual analog scale (VAS), comparing patients with peritoneal lavage (average pain score of 5) and those without lavage (average pain score of 5.9). The prevalence of pain greater than 6 was 30% in the group without lavage. Based on a 30% exposure frequency, a relative risk of three, a 95% confidence level, and 80% statistical power, it was determined that each group required 55 patients, maintaining a 1:1 ratio.

### 2.3. Sample

This study included 110 patients diagnosed with gallbladder pathology who underwent laparoscopic cholecystectomy at the General and Digestive Surgery Service of Hospital Nacional Edgardo Rebagliati Martins between July and December 2018. Before surgery, all patients scheduled for laparoscopic cholecystectomy were screened according to the predefined inclusion and exclusion criteria. After surgery, eligible patients were incorporated into the study through a systematic random selection process performed daily until the required sample size was reached. Participants were subsequently classified into two groups according to the surgeon’s routine intraoperative practice:

- Group 1: Patients who underwent peritoneal lavage after cholecystectomy.
- Group 2: Patients who did not undergo peritoneal lavage.

The exposure of interest (peritoneal lavage) was not assigned by the investigators and no randomization of the intervention was performed. Group allocation depended exclusively on the usual surgical decision-making of the operating surgeon. Approximately one or two exposed patients and one or two non-exposed patients were included per day until a total sample of 110 participants was achieved.

### 2.4. Inclusion Criteria

Adult patients with gallbladder pathology scheduled for elective laparoscopic cholecystectomy without intraoperative or postoperative complications.

### 2.5. Exclusion Criteria

Patients who received local anesthesia at the ports or intraperitoneal instillation of local anesthesia at the conclusion of the surgery.

### 2.6. Patient Selection Process

The surgical department has five departments, each with 12 surgeons and 24 hospital beds. For this study, we selected patients with a diagnosis of vesicular lithiasis scheduled for elective laparoscopic cholecystectomy, who were operated on by any of the surgeons in the Department of Surgery of the National Hospital Edgardo Rebagliati Martins (n = 60).

The assignment to study groups (with or without peritoneal lavage) was performed observationally after the surgical act, according to the intraoperative practice of the treating surgeon. It was observed that the use of peritoneal washing was not uniform among surgeons, and even among those who performed it, its application was not systematic in all cases. This approach reflects real-world clinical practice conditions. To partially reduce potential confounding related to this variability, the analyses were adjusted for relevant clinical and demographic variables.

### 2.7. Informed Consent Signature

On the day of the operation, patients signed a voluntary informed consent to participate in the study, awaiting their turn and entry into the operating room.

### 2.8. Peritoneal Lavage Procedure and Technique

In the operating room, peritoneal lavage was performed as follows:

- Patient Admission: The patient was admitted to the operating room and administered general anesthesia. The patient’s position was adjusted according to either the American technique (supine position) or the French technique (open legs).
- Preparation: Asepsis and antisepsis were performed using 4% chlorhexidine, followed by the placement of sterile drapes.
- Abdominal Access: A transumbilical incision was made. Pneumoperitoneum was established using either a 5 mm trocar (open) or a Veress needle (closed), with an intra-abdominal pressure of 15 mmHg. The trocars were introduced under direct vision, except for the first, which involved two 5 mm trocars and two 10 mm trocars. At the Hospital Nacional Edgardo Rebagliati Martins of the Peruvian Social Health Insurance System, the use of a 15 mm Hg pneumoperitoneum constitutes a routine surgical practice in laparoscopic procedures, particularly to optimize surgical field exposure and facilitate operative manipulation.
- Surgical Procedure: Existing peritoneal adhesions were released. The Calot’s triangle was dissected, identifying and clipping the cystic artery (1 clip) and cystic duct (3 clips). The proximal cystic artery was electrocauterized according to the routine standardized institutional surgical protocol used in this high-volume public hospital, which includes selective optimization of surgical clip utilization. The cystic duct was cut using Metzenbaum laparoscopic scissors. The gallbladder was freed from its bed using monopolar energy in a dissecting hook.
- Gallbladder Extraction: The gallbladder was placed in a sterile bag made from a surgical glove and temporarily placed over the liver.
- Peritoneal Lavage: The cholecystectomy was performed at the end by instillation of 0.9% saline solution at room temperature, using an irrigation cannula and intermittent suction. Irrigation was systematically directed to the subhepatic, vesicular, and perihepatic regions.

The volume of solution administered ranged from 400 to 700 mL (mean: 520.9 mL) with an average application time of 5 min. Although no strictly standardized protocol was used among all surgeons, these ranges reflect a relatively homogeneous intraoperative practice within the hospital. Given the observational real-world design of the study, minor variations in lavage volume and application time were allowed according to routine surgical practice. Nevertheless, future prospective studies with fully standardized lavage parameters may further enhance reproducibility and methodological consistency.

### 2.9. Data Collection Process

Before the start of the study, physicians and nurses responsible for data collection were trained in a standardized manner to ensure consistency in record-keeping.

Clinical-epidemiological data such as age, sex, body mass index, and other variables were obtained from the patients’ medical histories. In contrast, the evaluation of postoperative pain was carried out through direct interviews with patients, applied prospectively at the established times of the study.

Postoperative pain assessment was performed prospectively using the Visual Analog Scale (VAS) at predefined postoperative time points (6, 12, and 24 h), as described in the Outcomes subsection. The study was designed as a prospective observational analytical cohort, which ensured that the measurement of the main outcome (pain) was performed uniformly and in real time, minimizing record bias and ensuring comparability of data.

### 2.10. Pain Control Procedure

- Intraoperative Analgesia: All patients received intravenous Metamizole at a dose of 45 mg/kg body weight 30 min before the completion of surgery as part of the institutional analgesic protocol.
- Postoperative Analgesia: Postoperatively, Metamizole was administered every 8 h during the first 24 h. In cases of insufficient pain control, rescue analgesia consisting of 100 mg subcutaneous tramadol or 50 mg intravenous pethidine was administered on demand according to routine clinical practice. Pain assessment using the Visual Analog Scale (VAS) was performed at predefined postoperative time points independently of additional rescue analgesia administration.

### 2.11. Outcomes of the Study

The primary outcome of the study was postoperative pain intensity measured using the Visual Analog Scale (VAS). Pain assessments were performed prospectively at 6, 12, and 24 h after laparoscopic cholecystectomy by trained healthcare personnel using a standardized evaluation procedure. The VAS score ranged from 0 to 10, where 0 indicated no pain and 10 represented the worst imaginable pain.

The secondary outcome was the need for opioids or additional analgesic reinforcement during the first 24 postoperative hours. Rescue analgesia consisted of 100 mg subcutaneous tramadol or 50 mg intravenous pethidine administered according to routine clinical practice in cases of insufficient pain control.

### 2.12. Ethical Aspects

This study was conducted under the Declaration of Helsinki. The project was presented to the Institutional Ethics Committee for Human Research at Universidad Peruana Cayetano Heredia and was executed after approval (Approval number: 87-05-18, issued on 19 February 2018). Permission for study execution was also obtained from the Department of Surgery at the Edgardo Rebagliati Martins National Hospital. Patient data confidentiality was ensured by using codes instead of names to store information, and no identifying information was disclosed.

### 2.13. General Aspects of Statistical Analysis

Statistical analysis was performed using the R programming language (version 4.5). Numerical variables were described as means (standard deviation) or medians (interquartile range), as appropriate. Categorical variables were described as absolute frequencies and percentages.

### 2.14. Association Analysis Between Peritoneal Lavage and the Primary Outcome: Pain Level

To estimate the association of peritoneal lavage on pain levels, Linear Quantile Mixed Models (LQMMs) were fitted using the ‘lqmm’ package in R [18]. This approach was selected due to the nature of the primary study variable: pain level, measured using the Visual Analog Scale (VAS). This ordinal numeric variable has certain characteristics where increments across different parts of the scale may not represent the same change in pain experience [19,20]. To address this and provide a comprehensive view of pain distribution, quantile regressions were employed, focusing on the median, which offers a robust measure of central tendency in the presence of potential outliers [21]. Given the longitudinal nature of the data, as pain was measured at several time points, the assumption of independence between observations was challenged, and a certain correlation between repeated measures was expected. Therefore, LQMM, a longitudinal analysis strategy based on mixed models, was used to estimate both fixed and random effects in the quantiles of the response variable distribution. This choice captured the temporal dynamics inherent in our dataset [18,22].

Initially, a crude model evaluated the association between peritoneal lavage intervention and post-laparoscopy pain levels without adjusting for any other variables. Subsequently, an adjusted model accounted for potential confounding variables such as age, sex, body mass index (BMI), and height. Continuous variables (age, BMI, and height) were centered on their respective means to facilitate the interpretation of interaction coefficients and reduce multicollinearity. These variables were modeled continuously using B-splines with three degrees of freedom [23], to avoid the issues related to categorizing them [24,25].

For each model, 1000 bootstrap iterations were performed to obtain precise parameter estimates, and the ‘emmeans’ function [26] was used to provide point estimates and 95% confidence intervals for the fixed effects of the intervention (with peritoneal lavage vs. without peritoneal lavage) at each time point. To control the false discovery rate due to multiple testing, the Benjamini–Hochberg *p*-value adjustment method was applied. Results are presented as point estimates, 95% confidence intervals, and *p*-values. Error plots were also generated to visualize the median pain level and its 95% confidence intervals at each time point for each treatment group.

### 2.15. Association Analysis Between Peritoneal Lavage and the Secondary Outcome: Need for Opioids or Analgesic Reinforcement

For the statistical analysis, logistic regression models with Firth’s penalized maximum likelihood were used [27], due to their robustness in situations with small, poorly conditioned, or separated data [28]. Data were standardized by centering the variables for age, body mass index (BMI), and height on their means. A crude model was initially fitted, including only the variable of interest (peritoneal lavage). The relationship between peritoneal lavage and the need for opioids or additional analgesia was evaluated using likelihood ratios and Wald tests.

Next, a more comprehensive model was fitted, adding potential confounders such as age, sex, BMI, and height. As in the previous analysis, continuous numerical variables (age, BMI, and height) were centered on their arithmetic means, and the B-spline function with three degrees of freedom was used to capture possible non-linear relationships between these continuous variables and the outcome.

## 3. Results

### 3.1. Sample Characteristics

Between July 2018 and December 2018, 137 patients were invited to participate in the study. A total of 80.2% (110/137) agreed to participate, of which 75 (68.2%) were women. The participants’ ages ranged from 26 to 77 years, with a median age of 52.0 years (IQR: 42.3, 60.0). The patients’ body mass index (BMI) had a median value of 28.2 kg/m^2^ (IQR: 26.0, 32.0). A significant prevalence of overweight and obesity was observed in the study population. Specifically, 54 patients (49.1%) were classified as overweight, with a BMI between 25 and 29.9 kg/m^2^, and 38 patients (34.5%) were classified as obese, with a BMI of 30 kg/m^2^ or higher (Table 1).

Peritoneal lavage was performed on 50% of the patients, while the other half did not receive it. The groups were comparable, showing no significant differences in terms of age, sex, weight, height, or BMI (Table 2). No intra- or postoperative complications were reported in the patients included, according to the established selection criteria, which allowed for the evaluation of postoperative pain in a clinically homogeneous cohort.

### 3.2. Pain Level Progression

**Figure 1:**
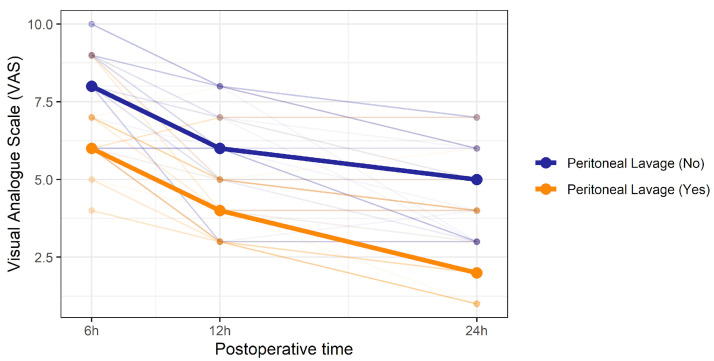
Individual and median pain trajectories among patients in this study. The spaghetti plot shows individual trajectories (thin, semi-transparent lines) and median values (thick, darkened lines) of pain levels according to their intervention group. Pain level was measured using the Visual Analogue Scale (VAS). Data were stratified according to the application of peritoneal lavage. Figure produced in software R v.4.4.1.

Regarding the progression of pain levels over 6, 12, and 24 h postoperatively, it was found that patients who received peritoneal lavage reported lower pain levels. At 6 h postoperatively, patients who underwent lavage reported a Visual Analog Scale (VAS) score of 6/10, while those who did not undergo lavage reported a VAS score of 8/10, with a highly significant difference (*p* < 0.001). Similar findings were observed at 12 and 24 h postoperatively. Figure 1 illustrates how pain levels consistently decreased over time in both groups; however, patients who received peritoneal lavage reported significantly lower pain levels compared to those who did not undergo lavage.

### 3.3. Association Between Peritoneal Lavage and Postoperative Pain Levels

**Figure 2:**
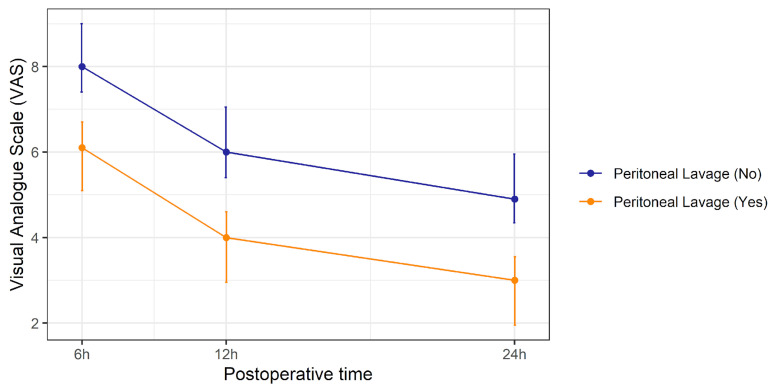
Adjusted median trajectories of pain level over time according to intervention groups. The plot shows a median and CI 95% values. Pain level was measured using the Visual Analogue Scale (VAS). Data were stratified according to the application of peritoneal lavage. Figure produced in software R v.4.4.1. CI 95%: Confidence Interval 95%.

Postoperative pain levels, judged by median differences, were different between the group of patients who received peritoneal lavage with saline solution and those who did not. Figure 2 provides a visual representation of the temporal evolution of adjusted median postoperative pain scores estimated through Linear Quantile Mixed Models (LQMMs), allowing readers to appreciate the trajectory, magnitude, and consistency of pain reduction over time between groups. The points represent adjusted median VAS scores, while the vertical bars indicate the corresponding 95% confidence intervals.

In contrast, Table 3 presents the detailed quantitative results of the LQMM analyses, including adjusted median differences, confidence intervals, and *p*-values. After adjustment for potential confounders, including sex, age, body mass index, and height, peritoneal lavage was consistently associated with significantly lower postoperative pain levels compared with the non-lavage group (*p* < 0.001). The adjusted median differences were −1.6 points (95% CI: −2.0 to −1.2) at 6 h, −1.7 points (95% CI: −2.1 to −1.2) at 12 h, and −1.6 points (95% CI: −2.1 to −1.1) at 24 h postoperatively, demonstrating a stable analgesic effect across all evaluated time points.

Additionally, Table 3 includes the crude multilevel model, which incorporates a random intercept to account for intraindividual correlation derived from repeated pain measurements obtained from each participant over time.

### 3.4. Association Between Peritoneal Lavage and the Need for Opioids or Analgesic Reinforcement

The effectiveness of the intervention was also explored in relation to the reduction in the need for opioids or analgesic reinforcement in the postoperative period. In the unadjusted model, an odds ratio (OR) of 0.38 (95% CI: 0.13 to 1.00) was observed for peritoneal lavage compared to the absence of lavage. After adjusting for confounding variables, the OR for peritoneal lavage intervention was 0.46 (95% CI: 0.13 to 1.51, *p* = 0.202), as shown in Table 4.

## 4. Discussion

The results of this study suggest that peritoneal lavage with saline solution is associated with significantly lower postoperative abdominal pain in patients undergoing laparoscopic cholecystectomy during the follow-up periods at 6, 12, and 24 h, compared to those who did not receive peritoneal lavage. The effectiveness of the intervention was similar across the three time points evaluated. However, no association was found between the effectiveness of the intervention and the reduction in the need for opioids or additional analgesic reinforcement in the postoperative period.

In our study, laparoscopic cholecystectomy was performed more frequently in women, with 75 (68.2%) of patients being female, which is consistent with the systematic review conducted by Xiong, in which between 52% and 88.5% were female patients [29]. It was also found that the BMI was within the overweight and obesity ranges (49.1% and 34.5%, respectively), but without significant differences between the two study groups.

When following up on the evolution of postoperative pain, it was observed that pain consistently decreased over time in both groups, but was significantly lower in the group that underwent peritoneal lavage with saline solution. This difference in the effectiveness of the intervention remained even after adjusting the model for variables such as sex, age, body mass index, and height, where peritoneal lavage was associated with a reduction in pain levels compared to the group that did not undergo the procedure. The difference in medians remained significantly lower in the group that received the peritoneal lavage intervention at all three time points evaluated: −1.6 points at 6 h, −1.7 points at 12 h, and −1.6 points at 24 h (*p* < 0.001).

These findings are clinically relevant and comparable with analgesic interventions described in the literature. For example, Ali et al. (2018) [30] demonstrated that intraperitoneal infiltration of bupivacaine significantly reduced postoperative pain measured using the analog visual scale (VAS) in patients undergoing laparoscopic cholecystectomy, particularly within the first 24 h. Although the mechanism of action differs, both approaches share a common goal: to modulate the local inflammatory response and decrease peritoneal irritation. In this context, our results suggest that peritoneal saline washing could offer a comparable analgesic effect through a physicochemical mechanism of cleaning the surgical bed, reduction in post-burn inflammation, and reduction in intra-abdominal inflammatory environment [30].

These findings support a potential association between peritoneal lavage and lower postoperative pain levels. Therefore, this procedure could facilitate tissue recovery following surgical injury, help clear inflammatory cytokines, promote the balance of the acidic environment in the intra-abdominal space, and consequently reduce postoperative pain.

Similar studies on the incidence of postoperative pain have reported that moderate to severe pain is more frequently observed at 6 h, with an average of 4.15/10 [31,32]. Few studies have performed saline instillation at the peritoneal level, with some reporting its use to reduce carbon dioxide absorption in these patients [14,16].

Also, reviews on the management of postoperative pain in laparoscopic cholecystectomy highlight the importance of multimodal strategies, including surgical interventions such as peritoneal washing. However, they also point out that the available evidence is heterogeneous and with limited standardization [17]. In this context, our results provide additional evidence by demonstrating a consistent effect on abdominal pain, specifically assessed using EVA, allowing for better comparison with contemporary studies.

Although there is research exploring the use of peritoneal irrigation as part of pain management, there are limited studies specifically analyzing its impact on early abdominal pain in elective laparoscopic cholecystectomies. particularly mediate application directed at the vesicular bed. Therefore, our findings reinforce the potential of peritoneal washing as a simple, reproducible, and low-cost intervention that could contribute to improving postoperative patient comfort.

There is significant interest in reducing postoperative pain, and numerous techniques have been described, such as the administration of high doses of opioids during surgery, which can lead to higher postoperative pain scores. In contrast, multimodal analgesia and preventive analgesia may reduce the need for opioids during surgery and mitigate postsurgical pain [31,33,34].

Non-opioid analgesics such as lidocaine, ketamine, and magnesium sulfate have shown evidence of being safe during the intraoperative period [33,35,36,37,38]. However, despite these efforts, the incidence of postoperative pain remains moderate to severe, as evidenced in our work, where the median pain score at 6 h after surgery remained at 8.0 points in those who received standard management without peritoneal lavage. This finding highlights the need for simple and low-cost complementary interventions, such as peritoneal washing, that can be easily integrated into multimodal analgesia protocols without increasing risks or costs definitively.

In this study, we also explored the effectiveness of the intervention concerning the reduction in opioid or analgesic reinforcement needs in the postoperative period. An OR of 0.46 (95% CI: 0.13 to 1.51, *p* = 0.202) was found in the group that underwent peritoneal lavage. This estimate suggests a trend toward reducing the need for additional analgesia in patients who underwent peritoneal lavage. Although this result did not achieve statistical significance, it is consistent with the trend observed in other studies where pain reduction does not always translate directly into lower opioid use. possibly due to standardized analgesic protocols or variability give the indication of analgesic rescue.

Considering that there is evidence associating higher BMI with increased postoperative pain, based on the fact that obesity and overweight are related to a persistent state of oxidation and inflammation, and that the surgical trauma accentuates the inflammatory process with greater cytokine release and consequently local pain or general discomfort [38,39], we analyzed this variable in our study and found no significant differences in BMI between the two study groups. Similarly, the evaluation of the effectiveness of our intervention and postoperative pain was adjusted for variables such as sex, age, body mass index, and height, thereby controlling for potential confounding variables.

Although the present study did not specifically evaluate shoulder tip pain, our findings are consistent with strategies aimed at reducing postoperative pain after laparoscopic surgery, in which evacuation of residual CO_2_ has been associated with earlier pain recovery [12,40]. Additionally, intraperitoneal infusion of normal saline has been described as facilitating the release of carbon dioxide through the laparoscopic ports [41,42]. However, other authors did not find clear benefits regarding shoulder tip pain [43,44], possibly because this type of pain is more closely related to diaphragmatic irritation caused by residual CO_2_ than to visceral peritoneal irritation, which was the primary pain outcome evaluated in our study.

The use of peritoneal lavage has also been reported at the end of laparoscopic gynecological procedures, with instillation of intraperitoneal fluids ranging from 1000 to 1500 mL of warm saline solution, where a lower incidence of shoulder pain was evidenced, with an OR of 0.67 and lower severity in the intervention group [45]. However, contradictory results have also been reported, where normal saline instillation was not effective in reducing shoulder pain in patients undergoing gynecological laparoscopic surgery [40], highlighting the need for further evidence.

Our findings are valuable because they show that peritoneal washing with saline is associated with a significant reduction in postoperative abdominal pain in patients undergoing laparoscopic cholecystectomy, besides being a simple and low-cost technique that can be easily replicated.

Although the role of peritoneal washing has been previously described within multimodal strategies for postoperative pain control, including recommendations from international guidelines such as PROSPECT/ESRA, that suggest the use of saline washing and aspiration as part of management to reduce pain after elective laparoscopic cholecystectomy [17,46], the available evidence comes from heterogeneous studies and does not always specifically evaluate abdominal pain as a primary outcome. In this context, our study provides additional clinical evidence by demonstrating a consistent effect of peritoneal washing on early abdominal pain, measured using EVA at defined times (6, 12 and 24 h), which suggests a better characterization of its magnitude of effect in a real practice scenario.

This study has some limitations that should be acknowledged. First, this was a single-center observational cohort study with a non-randomized design; therefore, the possibility of residual confounding and selection bias cannot be completely excluded. The decision to perform peritoneal lavage depended exclusively on the surgeon’s routine intraoperative practice rather than on a standardized allocation protocol.

Second, the intervention itself was not fully standardized across surgeons. Variations in lavage volume, irrigation technique, application time, and intraoperative surgical practices may have introduced technical heterogeneity that could influence postoperative pain outcomes. Although this variability reflects real-world clinical practice conditions and may increase external validity, it may also limit strict reproducibility of the intervention.

Third, surgeon-related factors, operative time, surgical complexity, and other intraoperative variables were not included in the adjusted statistical analyses. Consequently, the potential influence of these unmeasured variables on postoperative pain cannot be completely ruled out. Nevertheless, all procedures corresponded to elective uncomplicated laparoscopic cholecystectomies performed under relatively homogeneous institutional conditions using the same standardized pneumoperitoneum pressure (15 mmHg CO_2_).

Additionally, postoperative rescue analgesia was administered on demand according to routine clinical practice, which may have introduced variability in pain perception and assessment. However, pain measurements were obtained prospectively at predefined standardized postoperative time points by trained personnel.

Despite these limitations, the study provides clinically relevant real-world evidence regarding the association between peritoneal lavage and lower postoperative pain scores after elective laparoscopic cholecystectomy. Future multicenter randomized studies with fully standardized lavage protocols and adjustment for intraoperative surgical variables are warranted to confirm these findings.

Although the study data were prospectively collected in 2018, the statistical analyses and manuscript preparation were completed subsequently. Therefore, perioperative analgesic protocols, laparoscopic surgical practices, and postoperative pain management strategies may have evolved over time, potentially affecting the current generalizability of the findings. Nevertheless, the intervention evaluated in this study, peritoneal lavage with saline solution, remains a simple, low-cost, and broadly applicable intraoperative practice that continues to be used in contemporary surgical setting.

In conclusion, peritoneal lavage with isotonic saline at the end of laparoscopic cholecystectomy was associated with lower postoperative abdominal pain scores during the first 24 postoperative hours. These findings suggest a potential role for peritoneal lavage as a complementary strategy within multimodal postoperative pain management; however, prospective randomized studies are required to confirm causality and better establish its clinical effectiveness. However, given the limited available evidence and methodological heterogeneity, these findings should be interpreted with caution. Prospective studies with larger sample sizes and rigorous designs are required to confirm their effectiveness and establish their role within the standardized protocols of postoperative pain management.

## Figures and Tables

**Table 1:** Characteristics of the study population.

Characteristics	Total (n = 110)
Age (years)	
Median (IQR)	52.0 (42.3, 60.0)
Mean (SD)	50.8 (12.1)
Min, Max	26.0, 77.0
Sex (n,%)	
Male	35 (31.8)
Female	75 (68.2)
Weight (kg)	
Median (IQR)	72.5 (63.5, 83.9)
Mean (SD)	74.5 (14.4)
Min, Max	43.0, 119.0
Height (m)	
Median (IQR)	1.6 (1.5, 1.7)
Mean (SD)	1.6 (0.1)
Min, Max	1.5, 1.8
BMI (kg/m^2^)	
Median (IQR)	28.2 (26.0, 32.0)
Mean (SD)	29.1 (4.7)
Min, Max	17.9, 46.4
BMI categories	
Underweight (<18.5 kg/m^2^)	1 (0.9)
Normal (18.5–24.9 kg/m^2^)	17 (15.5)
Overweight (25–29.9 kg/m^2^)	54 (49.1)
Obesity (≥30 kg/m^2^)	38 (34.5)
Peritoneal lavage	
No	55 (50.0)
Yes	55 (50.0)

IQR: Interquartile range; SD: Standard Deviation; BMI: Body Mass Index.

**Table 2:** Basal characteristics of patients according to intervention.

Characteristics	Control (n = 55)	Peritoneal Lavage (n = 55)	*p*-Value
Age (years)			0.477 ^1^
Median (IQR)	49.0 (44.0, 57.5)	53.0 (40.5, 62.0)	
Mean (SD)	50.0 (11.9)	51.7 (12.4)	
Min, Max	26.0, 77.0	28.0, 72.0	
Sex (n,%)			0.838 ^3^
Male	17 (30.9)	18 (32.7)	
Female	38 (69.1)	37 (67.3)	
Weight (kg)			0.576 ^1^
Median (IQR)	72.9 (63.5, 84.2)	72.0 (63.8, 80.9)	
Mean (SD)	75.3 (15.8)	73.8 (13.1)	
Min, Max	43.0, 119.0	44.0, 106.0	
Height (m)			0.492 ^1^
Median (IQR)	1.6 (1.5, 1.6)	1.6 (1.5, 1.7)	
Mean (SD)	1.6 (0.1)	1.6 (0.1)	
Min, Max	1.5, 1.8	1.5, 1.8	
BMI (kg/m^2^)			0.190 ^4^
Median (IQR)	28.6 (26.5, 33.0)	27.6 (25.7, 30.5)	
Mean (SD)	29.5 (4.8)	28.6 (4.5)	
Min, Max	17.9, 38.2	20.6, 46.4	
BMI categories			0.338 ^2^
Underweight (<18.5 kg/m^2^)	1 (1.8)	0 (0.0)	
Normal (18.5–24.9 kg/m^2^)	9 (16.4)	8 (14.5)	
Overweight (25–29.9 kg/m^2^)	23 (41.8)	31 (56.4)	
Obesity (≥30 kg/m^2^)	22 (40.0)	16 (29.1)	

^1^ T-Student, ^2^ Fisher’s exact test, ^3^ Chi-squared test, ^4^ Wilcoxon rank-sum test.

**Table 3:** Estimates of Intervention Effectiveness on Pain Levels by Postoperative Time.

Raw Multivariate Model ^1^				Adjusted Multivariate Model ^2^
Time	Control (Estimated Median)	Peritoneal Lavage (Estimated Median)	Median Difference (CI 95%), *p*	Median Difference (CI 95%), *p*
6	8 (7.5–9)	6 (6–7)	−1.9 (−2.4 to −1.4), <0.001	−1.6 (−2 a −1.2), 1.6 × 10^−8^
12	6 (5–7.5)	4 (3–5)	−2 (−2.4 to −1.6), <0.001	−1.7 (−2.1 a −1.2), 1.1 × 10^−7^
24	5 (3–6)	2 (2–4)	−1.9 (−2.5 to −1.2), <0.001	−1.6 (−2.1 a −1.1), 6.2 × 10^−7^

CI 95% = Confidence Interval 95%. ^1^ Adjusted only for the random intercept effect, without adjustment for any potentially confounding fixed-effect variables. ^2^ Multivariate models with random effects. The patient was considered a random intercept, and the model was adjusted for potential confounding variables as fixed effects: sex, age, body mass index, and height.

**Table 4:** Estimates of intervention effectiveness and the need for opioids or analgesic reinforcement according to postoperative time.

	Raw Multivariate Model ^1^			Adjusted Multivariate Model ^2^		
	OR	CI 95%	*p*-Value	OR	CI 95%	*p*-Value
Intervention						
Peritoneal Lavage (No)	—	—		—	—	
Peritoneal Lavage (Yes)	0.38	0.13–1.00	0.050	0.46	0.13–1.51	0.202

CI 95% = Confidence Interval 95%; OR: Odds Ratio. ^1^ Adjusted only for the random intercept effect, without adjustment for any potentially confounding fixed-effect variables. ^2^ Multivariate models with random effects. The patient was considered as a random intercept, and the model was adjusted for potential confounding variables as fixed effects: sex, age, body mass index, and height.

## Data Availability

The original contributions presented in this study are included in the article/Supplementary Materials. Further inquiries can be directed to the corresponding authors.

## Supplementary Materials

The following supporting information can be downloaded at: https://www.mdpi.com/article/10.3390/medsci14020287/s1, Supplementary Table S1. STROBE Statement—checklist of items that should be included in reports of observational studies.
